# Mechanism of Exercise-Regulated Intestinal Flora for Alzheimer’s Disease Based on Gut–Brain Axis

**DOI:** 10.3390/nu18020254

**Published:** 2026-01-13

**Authors:** Huiying Zhao, Wei Wu, Xiaofan Men

**Affiliations:** 1 School of Exercise and Health, Shanghai University of Sports, Shanghai 200438, China; zhaohy1237@163.com; 2 School of Athletic Performance, Shanghai University of Sports, Shanghai 200438, China

**Keywords:** intestinal flora, gut–brain axis, Alzheimer’s disease, exercise

## Abstract

Alzheimer’s disease (AD) is a chronic neurodegenerative disorder characterized by progressive loss of cognitive function. Its main pathological features include accumulation of Amyloid-beta (Aβ) plaques, excessive phosphorylation of microtubule-associated protein tau (tau protein), and neuroinflammation. In recent years, studies have confirmed intestinal flora is closely connected to AD. Gut–brain axis has an important part in AD. Intestinal flora can achieve signal communication between gut and brain through metabolic, immune, neural, and endocrine pathways, thereby slowing down AD. It has been discovered that exercise is not only beneficial to physical health but also has a positive impact on the brain function. In recent years, more and more studies have found exercise can alleviate AD through the following four major pathways: regulating the diversity of intestinal flora, strengthening the blood–brain barrier (BBB), regulating immune homeostasis, and upregulating the brain-derived neurotrophic factor (BDNF). In this review, we have summarized intestinal flora in AD and systematically expounded potential regulatory pathways of exercise in modulating intestinal flora for AD. This provides a more theoretical basis for subsequent research targeting “gut–brain axis” to regulate AD. At the same time, this review also summarizes differences in different exercise types on improving intestinal flora for alleviating AD, providing new ideas and strategies for AD.

## 1. Introduction

Alzheimer’s disease (AD), as one of the most common neurodegenerative diseases worldwide, is characterized by progressive memory loss, cognitive decline, and abnormal neuropsychiatric behaviors [1]. The high disability rate and the long-term care demands resulting from AD have become major challenges for the global public health system, placing a significant strain on families and society [2]. With the acceleration of global aging population process, AD is showing a significant upward trend. WHO has listed it as a major health threat in the 21st century. Currently, the treatment methods for AD are still limited, and most treatments can only alleviate symptoms but fail to effectively stop the progression of the disease. There is an urgent need to explore new prevention and treatment strategies.

Gut–brain axis is central to the pathogenesis of AD. Systemic inflammatory response induced by microbiota imbalance will promote Amyloid-beta (Aβ) deposition and excessive phosphorylation of microtubule-associated protein tau (tau protein), aggravating neuroinflammation and exacerbating AD. The intestinal flora can achieve signal communication between gut and brain through metabolic, immune, neural, and endocrine pathways, thereby slowing down AD. It has been discovered that exercise not only benefits physical health but also exerts beneficial effects on brain function [3]. In recent years, numerous studies have found that exercise can alleviate AD through the following four major pathways: regulating the diversity of intestinal flora, strengthening the blood–brain barrier (BBB), regulating immune homeostasis, and upregulating the brain-derived neurotrophic factor (BDNF). Exercise can significantly improve AD symptoms, slow down disease progression, and have a positive effect on AD patients [4].

In this review, we have recapped the role of the intestinal flora in AD. To comprehensively evaluate the role of exercise-induced gut microbiota modulation in AD, this review was based on a literature search conducted primarily in PubMed and Web of Science databases. Relevant studies published in English were identified using combinations of keywords including “Alzheimer’s disease”, “exercise”, “physical activity”, “gut microbiota”, “intestinal flora”, and “gut–brain axis”. Both animal and human studies investigating the effects of physical exercise on gut microbiota and AD-related outcomes were considered. Studies not directly related to AD, lacking exercise interventions, or without relevance to gut microbiota regulation were excluded. The selected literature was analyzed to summarize gut microbiota alterations in AD, elucidate underlying regulatory mechanisms, and compare the effects of different exercise modalities. This provides more theoretical basis for subsequent research targeting “gutbrain axis” to regulate AD.

## 2. AD

AD is a neurodegenerative disease characterized mainly by progressive cognitive decline and memory loss, accounting for 60% to 80% of all cases of dementia [5]. The pathological features of AD include the deposition of Aβ plaques, neurofibrillary tangles, neuronal loss, and synaptic dysfunction [6].

As the population rapidly ages, AD is becoming more common every year. According to statistics, in 2020, approximately 50 million people worldwide suffered from dementia, among which AD was the most common type. It is projected that by 2050, the number of patients will exceed 150 million [7]. In China, with increase in life expectancy and aging, AD has been rising year by year and it has become a pressing public health issue that needs to be addressed [8]. The cause of AD is considered to be multifactorial, involving genetic factors, environmental factors, and the interaction of lifestyle factors [9]. Genetic studies have revealed that early-onset AD is closely associated with mutations in the amyloid-beta precursor protein and presenilin proteins (PSEN1 and PSEN2) genes, while late-onset AD is mainly related to the apolipoprotein E ε4 allele [10]. From the perspective of the pathogenesis, the amyloid protein cascade hypothesis posits that the imbalance between the production and clearance of Aβ protein is the core pathological process of AD [11]. Furthermore, the excessive phosphorylation of tau protein leads to the formation of neurofibrillary tangles, which is also a major factor contributing to neuronal death [1]. In recent years, neuroinflammation and dysbiosis of intestinal flora have been recognized as playing a significant role in AD [12].

Clinical diagnosis of AD mainly relies on collection of medical history, neuropsychological tests, and imaging examinations [13]. Nowadays, the detection of biomarkers, cerebrospinal fluid Aβ42 and tau levels, is also receiving increasing attention in clinical diagnosis [14]. The treatment strategies for AD include both drug therapy and non-drug interventions. However, current drugs merely offer transient symptomatic relief for AD. In 2021, US FDA approved the monoclonal antibody drug adunabucel for the treatment of Aβ. However, its effectiveness remains controversial [15]. These limitations underscore the urgent need for alternative or complementary strategies.

Given the multifactorial and systemic nature of AD, non-pharmacological interventions have attracted increasing interest [16]. Among them, physical exercise has emerged as a particularly promising approach. Exercise can regulate gut microbiota composition and diversity, thereby indirectly affecting central nervous system function through the gut–brain axis [17]. Accumulating evidence suggests that exercise can modulate neuroinflammation, enhance synaptic plasticity, improve cerebral blood flow, and influence metabolic and immune homeostasis [18]. Unlike single-target pharmacological therapies, exercise-based interventions simultaneously influence several key pathological pathways of AD, making them a valuable component in the long-term management and prevention of the disease.

## 3. Intestinal Flora and Gut–Brain Axis

### 3.1. Intestinal Flora

Intestinal flora refers to the collective term for the microbial communities present in the gastrointestinal tract. It is an important regulator of host health and its functions extend far beyond digestion and metabolism [19]. The microbial community passed vertically from the mother during infancy forms the initial microbiota. As the individual grows and the environment changes, it gradually stabilizes. The total number of bacteria in the adult’s intestinal tract exceeds 100 trillion [20]. The intestinal immune system selectively shapes the microbiota by secreting antimicrobial peptides and secreted immunoglobulin A. Meanwhile, the intestinal epithelial cells and the mucus layer they secrete provide physical support for the microbiota and limit its direct contact with the host [21]. Furthermore, the bile acids and digestive enzymes secreted by the host are also altering the competitive advantages of different bacterial communities.

The diversity of the intestinal flora is an important indicator for assessing intestinal health. Healthy intestinal flora usually has a high level of diversity, while in patients with AD, significantly reduced microbiota diversity has been observed [22]. The main components of the intestinal flora include Firmicutes phylum, Bacteroidetes, Actinobacteria phylum, and Proteobacteria. Firmicutes and Bacteroidetes dominate energy metabolism, breaking down complex polysaccharides in diet to produce short-chain fatty acids (SCFAs). The Actinobacteria assist in vitamin synthesis and bile acid metabolism, while the Proteobacteria play a complex role in intestinal homeostasis and inflammatory diseases [23]. In AD patients, lactobacilli and bifidobacteria in the gut are significantly reduced, while the abundance of Bacteroides, Proteus, and Clostridium increases [24]. This imbalance can lead to metabolic disorders and systemic inflammation in the host. Lactic acid bacteria and bifidobacteria have anti-inflammatory and immune-regulating effects. Their reduction will weaken the intestinal barrier function and exacerbate brain inflammation [25]. Vascular endothelial growth factor-C is downregulated by intestinal flora imbalance. Lipopolysaccharide (LPS) produced by Bacteroides and Proteus can induce the activation of microglia and neuroinflammation through the BBB, exacerbating the inflammatory response [26]. Clostridium destroys the BBB through exotoxins and directly acts on neurons with neurotoxins. The abnormal metabolites produced then activate glial cells, inducing and amplifying central nervous system inflammation. One study has found that transplanting intestinal flora from AD patients into germ-free mice leads to cognitive dysfunction and brain pathological features in the mice [27].

Host genes to some extent influence the composition of intestinal flora and even induce diseases. Although genetic factors are difficult to change, intestinal flora has been proven to be modifiable. The composition of intestinal flora is affected by various factors, including genetics, diet, lifestyle, and drug use [28]. The environment and diet are important exogenous factors in regulating intestinal flora. A diet high in fat and sugar has been proven to significantly reduce the diversity of flora and increase the abundance of harmful bacteria, while a high-fiber diet promotes beneficial bacteria proliferation [29]. A randomized controlled trial has shown that probiotic supplements can significantly improve the memory and executive function of patients with AD [30]. The Mediterranean diet and high-fiber diet can also help improve the pathological state of AD by increasing the abundance and metabolic activity of beneficial bacteria in the intestines [31]. Antibiotic treatment or fecal microbiota transplantation can also alter the composition of intestinal flora, significantly influencing Aβ deposition and neuroinflammation and improving symptoms of cognitive dysfunction [32]. Intestinal flora participates in multiple key physiological processes and carries profound clinical significance.

### 3.2. Gut–Brain Axis

Gut–brain axis, connecting gut and central nervous system, is a bidirectional communication network. Metabolites of intestinal flora can influence AD progression by regulating brain metabolism and neural functions [22]. In AD, the imbalance of intestinal flora leading to disruption of the gut–brain axis is a crucial link in the way intestinal flora affects brain function [33].

**Figure 1:**
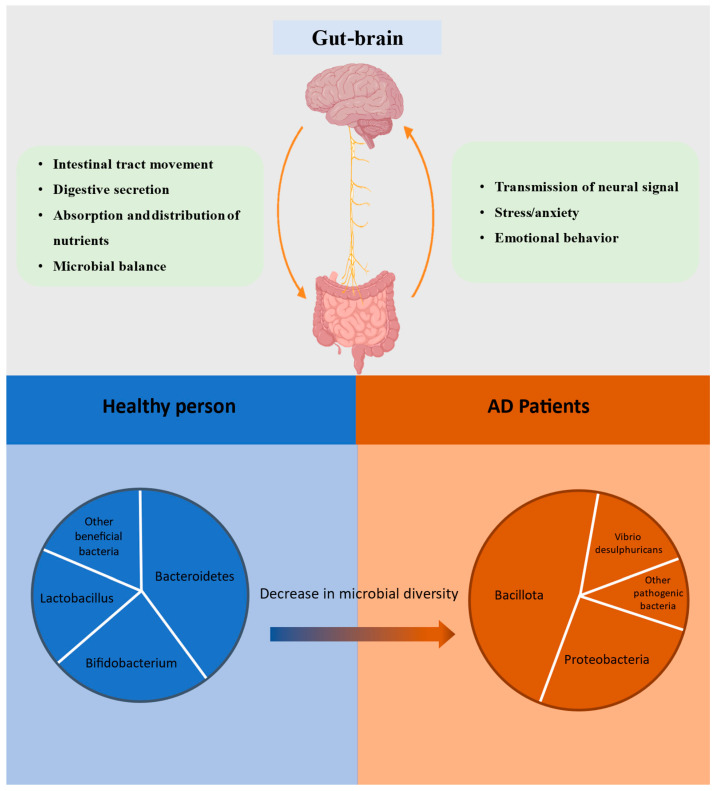
Gut–brain axis has a bidirectional regulatory effect. Brain can affect the environment of the intestines and the balance of the bacterial community. The imbalance of intestinal flora and disruption of intestinal environment can also exacerbate brain damage and aggravate cognitive impairment.

Vagus nerve is the primary communication pathway between intestines and central nervous system, capable of transmitting signals from the intestinal flora to the brain [32]. SCFAs produced by the metabolism of the intestinal flora can improve brain inflammation and cognitive function through the vagus nerve. Butyric acid among the SCFAs can also delay formation of neurofibrillary tangles by inhibiting the activity of histone deacetylase (HDAC) and reducing the acetylation sites of tau protein. In patients with AD, the levels of SCFAs were significantly reduced, thereby weakening their protective effect on the brain [34]. The intestinal flora also affects the inflammatory state of AD by regulating immune system. The disruption of the intestinal barrier allows LPS to enter the circulatory system, triggering a systemic inflammatory response. A study has shown LPS in the blood of AD patients increases, which can further exacerbate brain inflammation by activating microglial cells [35]. Meanwhile, the imbalance of microbiota leads to an increase in proportion of secondary bile acids. These secondary bile acids, through the TGR5-NF-κB signaling pathway, enhance the assembly of the NOD-like receptor thermal protein domain-associated protein 3 (NLRP3) inflammasome, thereby further amplifying the central inflammatory cascade [36]. Intestinal flora imbalance will downregulate vascular endothelial growth factor-C, weaken lymphatic drainage function of the meninges, and cause Aβclearance and tau proteins to be blocked. The disruption of the microbiota leads to the continuous stimulation of the intestinal mucosa by LPS, inducing interleukin-6 (IL-6) and tumor necrosis factor alpha (TNFα), thereby exacerbating inflammation [37]. The inflammatory signals reach the brain, activating the hypothalamic–pituitary–adrenal (HPA) axis. The adrenal glands continue to secrete more corticosterone, causing the hippocampus to be chronically exposed to high concentrations of glucocorticoids, leading to further decline in cognitive function [38] (Figure 1).

### 3.3. Potential Mechanisms by Which Gut Microbiota Regulate Alzheimer’s Disease

The intestinal flora achieves communication between gut and the brain mainly through metabolic, immune, neural, and endocrine pathways.

#### 3.3.1. Metabolic Mechanism

The intestinal flora breaks down dietary fibers and produces a large amount of SCFAs, mainly including butyric acid, propionic acid, and acetic acid [39]. SCFAs pass through the BBB via the bloodstream or by activating afferent fibers of vagus nerve, transmitting signals from intestinal wall to solitary nucleus, thereby influencing the brain [40]. SCFAs, especially butyric acid, can inhibit histone deacetylases, exerting a powerful anti-inflammatory effect, inhibit excessive activation of microglia, and reduce neuroinflammation [41]. SCFAs can effectively protect the BBB, maintain its integrity, and prevent harmful substances from the periphery from entering the brain [42]. Disorder of the intestinal flora can lead to a decrease in SCFAs and protective effect of neurotrophic factors on the brain will weaken, thereby exacerbating AD [43]. Butyric acid represents a potent inhibitor of histone deacetylase; when the dose of butyric acid is insufficient, the inhibition of HDAC is relieved. This leads to changes in the acetylation levels and activities of key kinases such as glycogen synthase kinase 3 beta (GSK-3β), directly promoting phosphorylation of tau protein at sites such as S396 and S404, thereby driving AD [44]. Propionic acid in SCFAs is an endogenous agonist for G-protein-coupled receptors (GPR41/43). A decrease in its level will downregulate the secretion of glucagon-like peptide-1 (GLP-1) by intestinal endocrine cells [45]. The reduction in GLP-1 will weaken the neural signals transmitted through the vagus nerve–solitary nucleus–hippocampus circuit, leading to a decrease in brain-derived neurotrophic factor in the hippocampus and ultimately damaging synaptic plasticity and learning and memory functions [46].

#### 3.3.2. Immunologic Mechanism

The intestine is the largest immune organ in human body, containing approximately 70% of immune cells [38]. The intestinal flora continuously interacts with the immune system and influences the brain through immune signals. A healthy microbiota helps maintain immune balance, while an imbalanced microbiota causes excessive activation of the immune system and the production of excessive pro-inflammatory cytokines [47]. When the intestinal flora is imbalanced, the number of Gram-negative pathogenic bacteria increases, and the release of their cell-wall component, LPS, also increases [48]. LPS and bacterial fragments enter the bloodstream, triggering a systemic low-level inflammation [49]. The inflammatory factors in the cycle can enter the brain through the damaged BBB or through brain regions lacking the BBB, activating TLR4-MyD88-NF-κB pathway, inducing NLRP3 inflammasome, and activating microglia cells [50]. The continuously activated microglia release a large amount of pro-inflammatory cytokines, driving neuroinflammation, and promoting Aβ deposition and tau protein phosphorylation, ultimately triggering neuronal injury and cognitive deterioration [51].

#### 3.3.3. Neural Mechanism

The neural pathway represents a rapid route of communication between the gut and the brain. Gut microbiota can synthesize, modulate, or influence a wide range of neuroactive compounds, including classical neurotransmitters and their precursors, such as serotonin (5-HT), dopamine, norepinephrine, γ-aminobutyric acid (GABA), acetylcholine, and histamine, as well as tryptophan metabolites and SCFAs [50]. These microbial-derived or microbiota-modulated neuroactive substances can act on the enteric nervous system and further influence central nervous system function through the circulation and the vagus nerve, thereby regulating mood, sleep, and cognitive processes. Disruption of gut microbiota homeostasis alters the production and balance of these neuroactive compounds, leading to the dysregulation of neural signaling along the gut–brain axis. The healthy functioning of the nervous system relies on the precise localization of water channel protein 4 (AQP4) at the footplates of astrocytes, which is known as “polarity distribution”, to efficiently drive fluid flow [52]. A study has shown that SCFAs, especially butyric acid, can help maintain and promote this polarity distribution of AQP4. When the intestinal flora is imbalanced, the number of beneficial bacteria decreases, resulting in a significant drop in SCFAs [40]. Metabolic wastes such as Aβ and tau proteins cannot be effectively processed, thus accumulating rapidly in the brain tissue, forming plaques and tangles, which further aggravates AD [53]. SCFAs themselves and the GABA precursors produced by the microbiota can affect the host’s sleep rhythm, and the function of neural–lymphatic system is most active during deep sleep [54]. Disorder of the intestinal flora can disrupt the sleep pattern, indirectly shortening the time for the brain to effectively remove waste [55].

#### 3.3.4. Endocrine Mechanism

**Figure 2:**
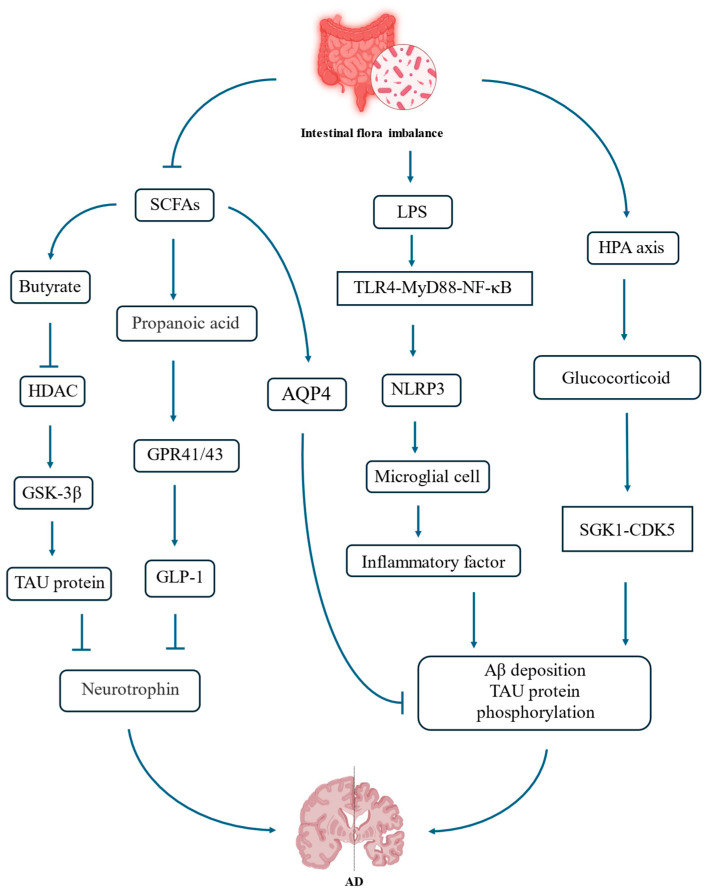
Intestinal flora regulates AD through multiple pathways, mainly involving metabolism, immunity, neurology, and endocrinology. Intestinal flora breaks down dietary fibers and produces SCFAs, which helps protect BBB and reduce neuroinflammation. When intestinal flora is imbalanced, inflammatory cells activate microglia, intensifying neuroinflammation. Intestinal flora can synthesize various neuroactive substances, effectively clearing the “garbage” in the brain. There is an HPA axis between intestine and brain. When intestinal flora is imbalanced, HPA is overly activated, exacerbating neuroinflammation and worsening AD.

The intestinal flora can affect human endocrine system. There is close two-way communication between the intestine and the brain, and HPA axis is the core hub. Psychological or physiological stress will activate the HPA axis, release glucocorticoids, and alter intestinal permeability and composition of microbiota [56]. Imbalance of intestinal flora is itself a kind of chronic stressor. An over-activated HPA axis can lead to long-term elevation of glucocorticoid levels [57]. High glucocorticoids have neurotoxicity, which can damage hippocampal neurons, increase BBB permeability, and promote neuroinflammation [58]. In the hippocampus, a crucial brain region, excessive glucocorticoids activate the SGK1-CDK5 signaling pathway through a mechanism dependent on glucocorticoid receptors, leading to the excessive phosphorylation of tau protein. This is one of the key pathological features of AD [59]. Long-term high levels of glucocorticoids can inhibit neurogenesis in the hippocampus and reduce the synthesis of synaptic-related proteins, directly leading to impaired memory function [60]. Furthermore, high-dose glucocorticoids will increase BBB permeability, promote neuroinflammation, and create conditions for harmful factors from the periphery to enter the brain [61] (Figure 2).

#### 3.3.5. Temporal and Causal Relationships Between Intestinal Flora and AD

Intestinal flora alterations precede, accompany, and reciprocally interact with Aβ and tau pathology across different stages of AD. In transgenic AD mouse models, reductions in microbial diversity and SCFAs-producing taxa have been observed prior to overt Aβ plaque deposition, indicating that dysbiosis may arise during preclinical disease stages rather than merely reflecting downstream neurodegeneration [62]. Harach and his research team confirmed in a 5 × FAD sterile mouse model that significant Aβ deposition could be detected in the hippocampus 3 weeks after feces from AD patients were transplanted, while the same-aged mice transplanted with feces from healthy controls did not develop plaques until 6 months later, suggesting that dysbiosis can “drive” rather than merely “coexist with” amyloidosis [63]. Overall, early or middle-aged intestinal flora imbalance will lower the threshold for the onset of Aβ and tau pathologies, and as the neurodegenerative process progresses, it will further disrupt the homeostasis of the intestine.

## 4. Exercise Modulates Intestinal Flora to Ameliorate AD

Regular exercise not only directly prevents and intervenes in AD, but recent studies have also revealed that its core function is largely achieved by regulating the “gut–brain axis” [64]. Exercise optimizes the intestinal flora structure, reduces anxiety and sleep disorders, regulates immune homeostasis, and upregulates neurotrophic factors, ultimately alleviating the core pathological processes of AD and delaying cognitive decline [65].

### 4.1. Reconstruct Composition and Diversity of Intestinal Microbiota

Exercise is a key factor for reshaping intestinal microecology and it significantly optimizes the bacterial community structure through various mechanisms. Regular exercise can significantly increase α diversity of intestinal flora, which is important for health and stability of the microecosystem [66]. This elevated α diversity is negatively correlated with the progression of AD, suggesting that a richer microbial repertoire slows cognitive decline. Regular exercise can significantly enhance the colonization and proliferation of beneficial bacteria such as Akkermansia, Bifidobacterium, and Lactobacillus, while inhibiting growth of opportunistic pathogens and restoring the ecological balance of the microbiota [67]. Akkermansia muciniphila can especially enhance the butyrate–intestinal barrier function, degrade mucin proteins, and provide living space for other beneficial bacteria. Its metabolic products can also regulate the immune system. Butyrate furthermore acts as a histone–deacetylase inhibitor, upregulating genes involved in amyloid clearance and downregulating BACE1, reducing AD pathology. Bifidobacteria and lactobacilli are both classic probiotics that can ferment dietary fibers to produce SCFAs, inhibit pathogens, and maintain the intestinal environment. The butyric acid-producing bacteria can convert dietary fibers into butyric acid, which is crucial for the brain. They also prevent harmful substances and inflammatory factors from entering the brain. Exercise can improve the local environment of the intestines. By promoting intestinal peristalsis, enhancing blood flow and oxygen supply, it creates favorable conditions for the survival of probiotics [68]. These clusters are consistently depleted in AD patients, which imply a direct impact in AD. At the same time, exercise also improves the overall insulin sensitivity of body and increases bacteria quantity associated with healthy metabolic profiles. Because brain insulin resistance is a recognized driver of AD, the microbiota-dependent improvement in systemic glucose tolerance forms an additional indirect pathway through which exercise protects cognition. A study has shown moderate-intensity regular exercise most powerfully and consistently improves intestinal flora, while excessive high-intensity training may temporarily disrupt the balance of the intestinal flora due to intense physiological stress [69].

### 4.2. Reduce Anxiety and Sleep Disorders

AD patients usually present with anxiety, depression, irritability, and sleep disorders [70]. Exercise can stimulate the brain to produce neurotransmitters such as endorphins, dopamine, and norepinephrine [71]. These neurotransmitters can naturally boost mood, induce a sense of pleasure, and effectively alleviate symptoms of anxiety and depression [72]. HPA axis is the core system for the body to respond to stress. Exercise can increase the production of butyric acid-producing bacteria, promote the increase in SCFA, and regulate the HPA axis [73]. Regular exercise can lower the basal cortisol level, enhancing the tolerance of AD patients to daily stress and thereby reducing irritability and anxiety. Moderate-intensity aerobic exercise for 6–12 weeks can increase the production of butyric acid-producing bacteria by 2–3 times, increase the concentration of butyric acid by 30–40%, reduce cortisol levels, and significantly alleviate anxiety-like behaviors [74]. A study found aerobic exercise (AE) significantly reduces the depression scores of AD patients by regulating HPA axis and promoting the release of endogenous opioids [75]. Sleep disorders are common symptoms in AD patients, and exercise exerts a strong beneficial impact on them. Exercise can improve sleep quality and enhance Aβ and tau protein by cerebrospinal fluid. Studies have shown engaging in low- to moderate-intensity exercise in the evening can increase the duration of deep sleep in AD patients, reduce the frequency of nighttime awakenings, and thereby improve overall sleep quality [76]. Exercise can promote the growth of bacteria such as Akkermansia and Romboutsia, enhance the expression of intestinal tryptophan hydrolase, increase 5-HT levels, prolong the duration of deep sleep, and reduce the number of nighttime awakenings [74].

### 4.3. Regulate Immune Homeostasis

Exercise regulates the immune system and improves the intestinal barrier, thereby bidirectionally modulating immune homeostasis. Regular exercise can increase the thickness of the intestinal mucus layer and strengthen the tight junctions between intestinal epithelial cells, forming a more robust physical barrier, effectively reducing entry of endotoxins [77]. LPS is a strong pro-inflammatory signal. Its reduction directly lowers the overall low-level inflammatory levels throughout the body, preventing excessive activation of peripheral immune cells and inflammatory factors overflow into brain. Exercise can induce the production of an adaptive state called “trained immunity” in innate immune cells, causing these cells to be in a more vigilant and finely regulated state [78]. When these cells migrate to the brain, they are less likely to be overly activated by pathological proteins such as Aβ, which helps to alleviate the chronic neuroinflammation in the AD brain and thereby creates a more protective neuroimmune environment [79]. Beyond the classical TLR4–NF-κB–NLRP3 inflammasome pathway, microglial polarization represents a critical neuro-immunological mechanism linking gut microbiota to AD progression. Although the M1/M2 framework provides a useful heuristic for describing broad inflammatory tendencies, accumulating evidence indicates that microglial activation in Alzheimer’s disease is highly heterogeneous and cannot be fully captured by a binary polarization model. Microglia exhibit functional plasticity, broadly categorized into a pro-inflammatory M1 phenotype and a neuroprotective, tissue-repairing M2 phenotype. In AD, chronic exposure to peripheral inflammatory signals and endotoxins such as LPS promotes sustained M1 polarization, thereby exacerbating synaptic loss, Aβ accumulation, and tau hyperphosphorylation. Recent transcriptomic and single-cell studies have further identified disease-associated microglia and related neurodegenerative phenotypes characterized by distinct immunometabolic programs, including altered lipid metabolism, phagocytic capacity, and mitochondrial function, which dynamically evolve during AD progression [80,81]. Exercise-induced modulation of gut microbiota significantly alters the availability of microbial metabolites, particularly SCFAs such as butyrate and propionate, which have been shown to reprogram microglial immunometabolism. These metabolites suppress NF-κB activation, inhibit NLRP3 inflammasome assembly, and promote a metabolic shift toward oxidative phosphorylation, thereby biasing microglial functional states toward neuroprotective and homeostatic profiles rather than strictly M1-/M2-defined phenotypes. Furthermore, indole-derived tryptophan metabolites produced by exercise-enriched microbiota activate the aryl hydrocarbon receptor signaling pathway in microglia, further reinforcing anti-inflammatory and neuroprotective phenotypes. Beyond inflammatory signaling, accumulating evidence suggests that exercise-induced modulation of gut microbiota also reshapes immune metabolism through the AMPK–PGC-1α axis. Microbial-derived metabolites, particularly SCFAs, can activate AMPK signaling in microglia and astrocytes, thereby promoting PGC-1α-dependent mitochondrial biogenesis and oxidative metabolism. This immunometabolic reprogramming shifts microglia away from a pro-inflammatory, glycolysis-dominated state toward a more oxidative, neuroprotective phenotype, enhancing energy efficiency and resilience in the AD brain. Through this mechanism, exercise-regulated microbiota indirectly supports neuronal and glial bioenergetics, linking peripheral microbial metabolism to central neuroimmune homeostasis. Collectively, these findings suggest that exercise regulates AD-related neuroinflammation not only by reducing inflammatory burden but also by actively reprogramming microglial functional states via the gut–brain axis.

### 4.4. Enhance Neural Activity of Brain

**Figure 3:**
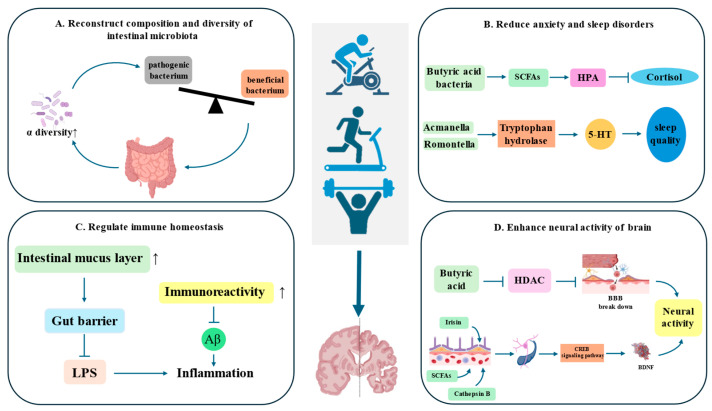
Exercise can regulate intestinal flora and improve AD. Exercise can optimize structure of intestinal flora and increase beneficial bacteria. Exercise can increase SCFAs and reduce anxiety and sleep disorders. Exercise can enhance the immune function of intestines and reduce inflammation. Exercise enriches the bacterial flora and further upregulates BDNF, alleviating AD.

Cognitive function decline is the core symptom of AD. Regular exercise can enhance neural activity and has a significant effect on improving the cognitive function of patients with AD [82]. The integrity of the BBB is the cornerstone for maintaining the internal environment of the central nervous system. Physical exercise enhances the function of the BBB, mainly through the metabolites produced by the induced microbiota [83]. The butyric acid produced during exercise can travel through the bloodstream to the brain and inhibit the activity of histone deacetylase (HDAC). When HDAC is inhibited, butyric acid further relaxes the chromatin structure, thereby upregulating tight junction proteins such as claudin-1 and occludin, which makes the structure between BBB endothelial cells more dense [84]. The enhanced BBB can effectively prevent inflammatory factors, neurotoxins, and some Aβ from entering the brain in the peripheral blood, creating a favorable environment for the brain and slowing down AD [85]. The hippocampus is the memory center of the brain and is one of the earliest and most severely affected areas in AD. Maintenance of hippocampus’ volume is directly related to the persistence of memory function. An animal experiment revealed that exercise not only enhanced the learning ability of mice, but also directly promoted new neurons in the hippocampus. The research conducted by Kirk I Erickson and others showed that healthy elderly individuals were divided into two groups. One group underwent AE for one year, while the other group received stretching and balance training. The results revealed that the volume of the anterior part of the hippocampus in the AD patients of the AE group increased by approximately 2%. In contrast, the volume of the hippocampus in the AD patients of the control group decreased by about 1.4% due to normal aging [77]. This process is closely related to the increase in BDNF levels [86]. BDNF is a key molecule that supports neurons’ survival, synaptic plasticity, and learning and memory. BDNF can promote growth, survival and differentiation of neurons, and enhance synaptic plasticity [87]. Exercise can directly stimulate skeletal muscles to release muscle factors such as irisin and cathepsin B [88]. Muscle factors can cross BBB and directly act on hippocampal and cortical neurons, activating the CREB signaling pathway, thereby strongly upregulating BDNF [89]. SCFAs produced by exercise, especially butyric acid, can cross BBB and significantly enhance the upregulation effect of exercise on BDNF [90]. Chronic neuroinflammation can inhibit the expression of BDNF. Exercise can effectively reduce the inflammatory environment in the brain and alleviate AD [91] (Figure 3).

How far these benefits extend into the earliest, metabolite-driven phase of AD pathology is now beginning to be quantified. At the metabolite-biomarker level, a recent longitudinal multi-omics review indicates that activation of the tryptophan–kynurenine pathway and accumulation of secondary bile acids correlate dose-dependently with tau phosphorylation, and that these microbial metabolite shifts typically antedate Aβ-PET positivity or measurable hippocampal atrophy [50]. Whether exercise-induced increases in butyrate or blockade of the kynurenine axis can actually reverse these biomarker trajectories remains untested in randomized trials. Current evidence is therefore still at the “hypothesis-association” stage and awaits confirmation through longitudinal, multi-omic intervention studies.

## 5. Analysis of Different Exercise on Improving Intestinal Flora for Alleviating AD

Exercise can be broadly classified according to intensity, metabolic characteristics, and primary physiological targets. In the context of Alzheimer’s disease and gut microbiota modulation, AE, resistance training (RT), and high-intensity interval training (HIIT) are the most frequently investigated modalities in both animal and clinical studies. These exercise types differ substantially in energy metabolism, muscular involvement, and systemic stress responses, thereby exerting distinct effects on gut microbiota composition and gut–brain axis signaling. AE, typically performed at moderate intensity over a sustained duration, primarily relies on oxidative metabolism and has been consistently associated with improvements in cardiovascular function, intestinal motility, and microbial diversity. RT is characterized by repeated muscle contractions against external loads and mainly influences metabolic regulation, muscle-derived myokine secretion, and insulin sensitivity. HIIT consists of brief bouts of vigorous exercise interspersed with recovery periods and is notable for its high metabolic demand and strong mitochondrial and stress-adaptive effects. These three exercise modalities were therefore selected in this review because they represent distinct and complementary physiological pathways through which physical activity may regulate gut microbiota and, in turn, influence Alzheimer’s disease-related pathology via the gut–brain axis.

### 5.1. AE

AE is the most widely studied and evidence-supported form of exercise, and it has shown positive benefits in improving the cognitive function of AD patients [92]. AE appears particularly effective in the early and prodromal stages of AD by stabilizing intestinal flora composition, enhancing short-chain fatty acid production, and reducing systemic low-grade inflammation [93]. The mechanism by which AE improves intestinal flora and subsequently alleviates AD focuses on optimizing the internal environment of the intestines [64]. AE can effectively promote intestinal peristalsis, shorten the intestinal transit time, and prevent the excessive proliferation of harmful bacteria [94]. Meanwhile, AE improves the circulation of blood throughout the body and the supply of oxygen, providing more sufficient nutrients and oxygen to the intestinal epithelial cells, creating an environment conducive to survival of aerobic or facultative anaerobic beneficial bacteria such as Akermania and Bifidobacterium [84]. Long-term and moderate-intensity aerobic exercise is generally regarded as the most effective way to increase the α diversity of intestinal flora [95]. A randomized controlled trial demonstrated that after 12 months of moderate-intensity AE intervention, the memory and executive function of AD patients showed significant improvement [96]. Furthermore, AE can significantly increase the abundance of the bacterial community with anti-inflammatory and barrier-protection functions [97]. These beneficial bacterial changes led to an increase in butyric acid levels, strengthened BBB, and directly exerted anti-inflammatory effects [85]. AE can effectively improve the systemic circulation and optimize the local blood supply and peristalsis of the intestinal tract. This provides a healthier internal environment for the microbiota and directly helps to strengthen the physical barrier of the intestine, reducing LPS into the bloodstream [98]. The advantage of AE lies in its comprehensiveness and stability. By optimizing the microbial ecosystem, it lays a healthy and stable foundation for the gut–brain axis [99].

### 5.2. RT

Unlike AE, which mainly acts on the intestinal environment, the core mechanism by which RT alleviates intestinal flora and improves AD lies in its active regulation of the body’s metabolism [100]. RT effectively improves glucose metabolism and insulin sensitivity by increasing muscle mass and promotes the proliferation of bacteria associated with healthy metabolic profiles [101]. After RT, changes in microbiota functional modules related to branched-chain amino acid metabolism can be observed [102]. RT exerts stronger effects during intermediate stages of AD through skeletal muscle-derived myokines, such as irisin, which modulate neuroinflammation and promote synaptic plasticity via the gut–brain axis [103]. Irisin can directly penetrate the BBB and increase BDNF, thereby promoting nerve regeneration [104]. Furthermore, the study also found that irisin can promote the polarization of microglia towards an anti-inflammatory phenotype and participate in clearance of Aβ [105] by correcting the disorder of the insulin signaling pathway in the brain and thereby improving the energy metabolism of neurons and synaptic functions [106]. A study found that AD patients who performed resistance exercises three times a week had significantly higher cognitive function scores compared to the control group and were able to significantly improve cognitive function of AD patients [107].

### 5.3. HIIT

HIIT is renowned for its high time efficiency. However, the intense physiological stress it causes has a dual impact on the microbiome and AD. The effect highly depends on an individual’s adaptability and training program [108]. Moderate HIIT has been proven to rapidly improve cardiovascular metabolic health and may also bring about beneficial changes in the microbiome. Its unique mechanism advantage lies in its ability to efficiently improve mitochondrial function [109]. HIIT can activate AMPK/PGC-1α signaling pathway, promoting brain energy metabolism and mitochondrial biosynthesis, which is crucial for neurons with extremely high energy demands [89]. For individuals who can adapt well, the moderate stress induced by HIIT can enhance the body’s antioxidant and anti-inflammatory capabilities and exert a long-term inhibitory effect on neuroinflammation. However, due to its extremely high intensity, improper or excessive HIIT may cause a strong sympathetic–adrenal stress response, resulting in a temporary increase in intestinal blood flow and permeability. This, in turn, can have adverse effects on the balance of microbiota and the intestinal barrier [110]. Even though HIIT provides metabolic and mitochondrial benefits in selected individuals, it requires careful individualization due to its potential to transiently disrupt intestinal barrier integrity [111]. The dual effects of HIIT can be best interpreted through the concept of exercise-induced hormesis. Within an optimal hormetic window, transient metabolic and oxidative stress activates adaptive signaling pathways, including AMPK–PGC-1α and Nrf2, thereby enhancing mitochondrial biogenesis, antioxidant capacity, and neuroprotection [112]. However, when exercise intensity or frequency exceeds the individual’s adaptive capacity, excessive sympathetic activation and glucocorticoid release may disrupt intestinal perfusion and epithelial integrity, increase gut permeability, and promote systemic inflammation. This tipping point marks the transition from neuroprotective hormesis to pro-inflammatory stress, underscoring the importance of individualized HIIT prescriptions in vulnerable populations such as AD patients. For elderly patients with weak health conditions or those with obvious cognitive impairments, such as those with AD, the risks are higher and it needs to be carried out under professional guidance and strict monitoring [113].

Different exercises regulating intestinal flora and alleviating AD are all detailed in Table 1.

## 6. Discussion

Although exercise can improve AD, its beneficial effect on the progression of AD cannot be solely attributed to the regulation of gut microbiota. Exercise involves the combined action of the endocrine function of skeletal muscles, immune regulation, metabolic homeostasis, and the communication between the gut and the brain through a complex network. Skeletal muscle should be regarded as a central endocrine organ linking exercise to gut–brain axis regulation in AD. Muscle contraction induces the release of multiple myokines, including irisin, cathepsin B, and IL-6, which exert systemic effects on immune modulation, intestinal barrier integrity, and neurotrophic signaling. These myokines can directly or indirectly influence gut microbiota composition and metabolic function, thereby amplifying exercise-induced neuroprotection beyond mechanical or cardiovascular adaptations.

Biological sex represents an important yet underexplored modifier of the exercise–microbiota–brain axis in AD. Epidemiological data indicate that women exhibit a higher lifetime risk and faster progression of AD, which has been partially attributed to sex-specific hormonal, immunological, and metabolic differences. Emerging evidence suggests that gut microbiota composition and diversity differ significantly between males and females, influencing immune tone, energy metabolism, and neuroinflammatory susceptibility. Exercise-induced metabolic adaptations also display sex-dependent patterns. For instance, estrogen signaling has been shown to interact with SCFA-mediated anti-inflammatory pathways, potentially amplifying the neuroprotective effects of exercise in females. Conversely, males may exhibit stronger muscle-derived myokine responses, such as irisin release, which differentially modulate BDNF expression and mitochondrial function. Future studies should incorporate sex-stratified analyses to refine exercise prescriptions and microbiota-targeted interventions for AD prevention and treatment.

## 7. Conclusions and Perspectives

The intestinal flora and its metabolites have a profound impact on AD. The intestine, as a highly complex metabolic organ within the human body, and the gut microbiota within it participate in the pathogenesis of AD at multiple levels through the bidirectional communication pathway of the gut–brain axis. Once the intestinal ecosystem becomes imbalanced, it will exacerbate the onset of AD and aggravate cognitive dysfunction. Exercise not only directly improves AD but can also enhance AD by regulating intestinal flora. Exercise, as an effective non-pharmacological intervention method, has positive effects on AD in multiple aspects. This review elaborates on the potential mechanisms by which exercise regulates intestinal flora to improve AD from the following four aspects: optimizing the structure of intestinal flora, strengthening the BBB, regulating immune homeostasis, and upregulating BDNF. At the same time, this review also conducts a differential analysis of the effects of different exercise types on improving intestinal flora for alleviating AD.

Physical exercise emerges as a promising non-pharmacological intervention capable of modulating AD pathology both directly and indirectly through the regulation of gut microbiota. Despite these advances, the molecular and cellular networks linking exercise, gut microbiota, and AD remain incompletely understood. Future research should prioritize large-scale human clinical trials combined with mechanistic animal studies to characterize microbiota structural and functional differences between AD patients and cognitively healthy individuals. Integrating multi-omics approaches—including metagenomics, metabolomics, and transcriptomics—will be essential for identifying AD-specific microbial signatures and metabolic biomarkers. Looking ahead, individualized exercise prescriptions, optimization of training intensity and duration, and combined strategies incorporating probiotic or dietary interventions represent key directions for future investigation. Through systematic clinical validation and long-term follow-up studies, it is anticipated that exercise-based modulation of the gut–brain axis may provide more precise, effective, and accessible strategies for AD prevention and treatment, ultimately improving patient quality of life and reducing the societal burden of the disease.

## Figures and Tables

**Table 1:** Different exercises regulate intestinal flora to alleviate AD.

Types	Key Points of Regulation	Impact on Intestinal Flora	Potential Benefits for AD	Ref.
AE	Optimize the internal environment of the intestines	Increase α diversity; Enhance beneficial bacteria (Acinetobacter and Bifidobacterium); Promote butyric acid production	Improve cognitive function; Anti-inflammation; Strengthen intestinal and blood–brain barriers	[77,84,85,93,94,95,96,97,98,99]
RT	Improve overall metabolism	Promote bacteria growth related to healthy metabolism; Modify metabolic function of branched-chain amino acids	Improve insulin resistance in brain; Up-regulate BDNF; Promote Aβ clearance	[100,101,102,103,104,105,106,107]
HIIT	Efficiently improve mitochondrial function	Lead to beneficial changes in metabolism-related bacterial flora; Improper training carries negative risks	Rapidly improve brain energy metabolism; Inhibit neuroinflammation long-term	[90,108,109,110]

## Data Availability

No new data were created or analyzed in this study. Data sharing is not applicable to this article.
